# Exploring the Gap Between Excess Mortality and COVID-19 Deaths in 67 Countries

**DOI:** 10.1001/jamanetworkopen.2021.17359

**Published:** 2021-07-16

**Authors:** Francesco Sanmarchi, Davide Golinelli, Jacopo Lenzi, Francesco Esposito, Angelo Capodici, Chiara Reno, Dino Gibertoni

**Affiliations:** 1 Department of Biomedical and Neuromotor Science, Alma Mater Studiorum–University of Bologna, Bologna, Italy

## Abstract

This cross-sectional study examines the difference between COVID-19 confirmed mortality and excess mortality in 67 countries.

## Introduction

During the SARS-CoV-2 pandemic, a surge in overall deaths has been recorded in many countries, most of them likely attributable to COVID-19. However, COVID-19 confirmed mortality (CCM) is considered an unreliable indicator of COVID-19 deaths because of national health care systems’ different capacities to correctly identify people who actually died of the disease.^1,2^ Excess mortality (EM) is a more comprehensive and robust indicator because it relies on all-cause mortality instead of specific causes of death.^3^ We analyzed the gap between the EM and CCM in 67 countries to determine the extent to which official data on COVID-19 deaths might be considered reliable.

## Methods

In this cross-sectional study, we retrieved aggregated country-level data on population and COVID-19 overall confirmed cases, deaths, and testing as of December 31, 2020, from Our World in Data. Data on countries’ overall deaths from 2015 to 2020 were obtained from the World Mortality Data set (eAppendix in the Supplement). This research was based on public use datasets that do not include identifiable personal information and, per the Common Rule, was exempt from Institutional Review Board review and approval. For the same reason, no informed consent was required. This study follows the Strengthening the Reporting of Observational Studies in Epidemiology (STROBE) reporting guideline.

Negative binomial regression models were used to estimate projected deaths in 2020 using mortality data from 2015 to 2019. Two-sided 95% CIs for country-specific projected deaths were calculated applying the normal approximation to the Poisson distribution. EM in the pandemic period (ie, February 26 to December 31, 2020) was estimated as the difference between cumulative observed deaths and projected deaths. Countries’ testing capacity was assessed with their cumulative test-to-case ratio (eAppendix in the Supplement). The association between country-specific cumulative CCM and EM per 100 000 population of 2020 was displayed using a scatterplot, in which the identity line discriminates countries with EM exceeding CCM from those with EM lower than CCM. A color was assigned to countries based on their decile of testing capacity. All analyses were performed using R version 4.0.4 (R Project for Statistical Computing). Details on the analytic approach are available in the eAppendix in the Supplement.

## Results

Most of the 67 countries experienced an increase in mortality during 2020 (Table). Among countries with increased mortality (ie, those located above 0 on the y-axis in the Figure), a small number appeared under the identity line, showing lower-than-expected mortality after subtracting COVID-19 deaths. Countries located above the identity line can be visually classified into 2 groups: 1 with several Latin American and East European countries, which exhibit a large gap between EM and CCM (eg, Mexico, 212 excess deaths vs 96 COVID-19 deaths per 100 000 population); the other, more heterogeneous group showed a moderate EM beyond CCM (eg, Greece, 57 excess deaths vs 45 COVID-19 deaths per 100 000 population). Countries with negative EM also had very low CCM and were mainly located in East Asia. The lowest figures of EM and CCM generally belonged to countries with higher testing capacity (in green) and the largest differences between EM and CCM to countries with poorer testing capacity (in red).

**Table.** Excess Deaths and Test-to-Case Ratio, February 26 to December 31, 2020, 67 Countries

Country	Observed deaths	Projected deaths (95% CI)	Ratio of observed to projected	Excess deaths per 100 000	Total COVID-19 deaths	COVID-19 deaths per 100 000	Excess deaths attributed to COVID-19, %^a^	Test to case ratio
Albania	23 400	18 154 (17 572-18 736)	1.29	182.29	1181	41.04	23	4.24
Australia	119 924	124 531 (121 707-127 355)	0.96	−18.07	909	3.56	NA	396.15
Austria	75 588	67 431 (66 021-68 841)	1.12	90.57	6059	67.27	74	10.63
Belgium	108 160	89 308 (87 083-91 533)	1.21	162.66	19 361	167.05	103	10.77
Bolivia	69 752	44 655 (44 586-44 724)	1.56	215.00	9165	78.51	37	2.59
Brazil	1 385 572	1 139 346 (1 135 039-1 143 653)	1.22	115.84	194 949	91.72	79	1.42
Bulgaria	105 383	88 056 (86 220-89 892)	1.20	249.37	7405	106.57	43	5.68
Chile	109 238	95 428 (92 487-98 369)	1.14	72.24	16 488	86.25	119	10.59
Colombia	255 360	210 524 (208 704-212 344)	1.21	88.12	42 620	83.76	95	4.93
Costa Rica	22 135	21 321 (20 940-21 702)	1.04	15.98	2185	42.89	268	2.53
Croatia	47 865	42 092 (41 322-42 862)	1.14	140.62	3795	92.44	66	4.83
Cyprus	5256	4994 (4842-5146)	1.05	29.91	117	13.36	45	47.12
Czechia	109 308	93 318 (91 664-94 972)	1.17	149.31	11 302	105.54	71	Missing
Denmark	45 582	45 673 (44 789-46 557)	1.00	−1.57	1226	21.17	NA	64.20
Ecuador	102 468	63 902 (63 241-64 563)	1.60	218.59	14 001	79.36	36	3.30
Estonia	13 356	12 858 (12 610-13 106)	1.04	37.54	221	16.66	44	22.81
Finland	46 142	45 369 (44 587-46 151)	1.02	13.95	550	9.93	71	69.39
France	561 871	507 513 (497 166-517 860)	1.11	79.76	64 203	94.21	118	Missing
Georgia	41 771	37 461 (36 571-38 351)	1.12	108.04	2505	62.79	58	Missing
Germany	822 155	793 924 (775 602-812 246)	1.04	33.69	32 267	38.51	114	21.20
Greece	107 886	101 976 (100 065-103 887)	1.06	56.70	4730	45.38	80	24.36
Guatemala	81 804	71 611 (71 075-72 147)	1.14	56.89	4781	26.69	47	4.43
Hungary	118 424	105 853 (103 646-108 060)	1.12	130.13	9292	96.19	74	6.91
Iceland	1889	1903 (1860-1946)	0.99	−4.10	29	8.50	NA	41.96
Israel	40 261	37 288 (36 438-38 138)	1.08	34.35	3292	38.03	111	19.81
Italy	630 694	521 949 (511 176-532 722)	1.21	179.86	73 019	120.77	67	12.62
Japan	1 131 879	1 171 088 (1 154 918-1 187 258)	0.97	−31.00	3286	2.60	NA	19.03
Kazakhstan	139 904	109 835 (108 318-111 352)	1.27	160.14	2761	14.70	9	27.53
Kyrgyzstan	33 995	27 135 (27 045-27 225)	1.25	105.15	1355	20.77	20	Missing
Latvia	23 869	23 159 (22 643-23 675)	1.03	37.64	603	31.97	85	21.44
Lithuania	36 750	30 847 (30 277-31 417)	1.19	216.84	1695	62.26	29	11.57
Luxembourg	3960	3664 (3565-3763)	1.08	47.29	489	78.12	165	35.58
Malaysia	145 604	150 442 (150 192-150 692)	0.97	−14.95	471	1.46	NA	29.59
Malta	3311	3032 (2928-3136)	1.09	63.19	215	48.69	77	40.51
Mauritius	9250	9595 (9540-9650)	0.96	−27.13	10	0.79	NA	Missing
Mexico	898 733	625 345 (616 114-634 576)	1.44	212.04	123 845	96.05	45	2.40
Moldova	34 043	29 276 (28 381-30 171)	1.16	118.17	2985	74.00	63	Missing
Mongolia	13 258	14 554 (14 494-14 614)	0.91	−39.53	1	0.03	NA	492.61
Montenegro	6141	5455 (5319-5591)	1.13	109.22	677	107.79	99	Missing
Netherlands	141 911	126 826 (124 163-129 489)	1.12	88.04	11 305	65.98	75	6.69
New Zealand	27 643	29 907 (29 211-30 603)	0.92	−46.95	25	0.52	NA	650.26
North Macedonia	21 622	16 537 (16 197-16 877)	1.31	244.07	2503	120.14	49	4.83
Norway	33 544	33 460 (32 815-34 105)	1.00	1.55	433	7.99	515	56.91
Oman	9072	7782 (7726-7838)	1.17	25.26	1499	29.35	116	Missing
Panama	20 313	17 527 (17 305-17 749)	1.16	64.57	4022	93.21	144	5.28
Paraguay	28 707	27 376 (27 239-27 513)	1.05	18.66	2262	31.71	170	5.19
Peru	192 215	107 608 (106 057-109 159)	1.79	256.60	37 525	113.81	44	3.43
Poland	407 017	343 727 (337 185-350 269)	1.18	167.23	27 454	72.54	43	5.36
Portugal	104 427	90 907 (88 257-93 557)	1.15	132.59	6751	66.21	50	13.73
Qatar	2237	1882 (1869-1895)	1.19	12.32	245	8.50	69	8.63
Romania	251 366	214 243 (209 476-219 010)	1.17	192.97	15 469	80.41	42	7.61
Russia	1 817 225	1 460 074 (1 433 045-1 487 103)	1.24	244.73	56 271	38.56	16	29.14
Serbia	97 126	83 772 (82 148-85 396)	1.16	196.25	3211	47.19	24	6.80
Singapore	18 157	18 382 (18 363-18 401)	0.99	−3.85	29	0.50	NA	92.71
Slovakia	49 240	44 053 (43 267-44 839)	1.12	95.01	1983	36.32	38	18.10
Slovenia	20 034	17 033 (16 630-17 436)	1.18	144.35	2631	126.56	88	5.55
South Korea	252 127	252 686 (249 165-256 207)	1.00	−1.09	869	1.69	NA	65.46
Spain	417 857	339 985 (332 077-347 893)	1.23	166.55	50 442	107.89	65	11.77
Sweden	80 125	71 487 (69 939-73 035)	1.12	85.53	8279	81.98	96	Missing
Switzerland	64 126	55 415 (54 275-56 555)	1.16	100.65	7493	86.58	86	8.13
Taiwan	142 272	147 889 (145 095-150 683)	0.96	−23.58	6	0.03	NA	158.93
Thailand	414 555	414 290 (412 595-415 985)	1.00	0.38	63	0.09	24	228.14
Tunisia	61 509	59 078 (57 198-60 958)	1.04	20.57	4570	38.67	188	Missing
Ukraine	516 097	476 463 (466 623-486 303)	1.08	90.63	19 281	44.09	49	5.19
United Kingdom	576 821	494 271 (481 999-506 543)	1.17	121.60	71 675	105.58	87	21.06
United States	2 870 292	2 419 814 (2 387 664-2 451 964)	1.19	136.09	344 730	104.15	77	12.70
Uzbekistan	150 808	133 298 (128 228-138 368)	1.13	52.32	614	1.83	4	Missing

Abbreviation: NA, not applicable.

^a^ Excess deaths attributable to COVID-19 calculated by dividing COVID-19 deaths per 100 000 by excess deaths per 100 000.

**Figure.**
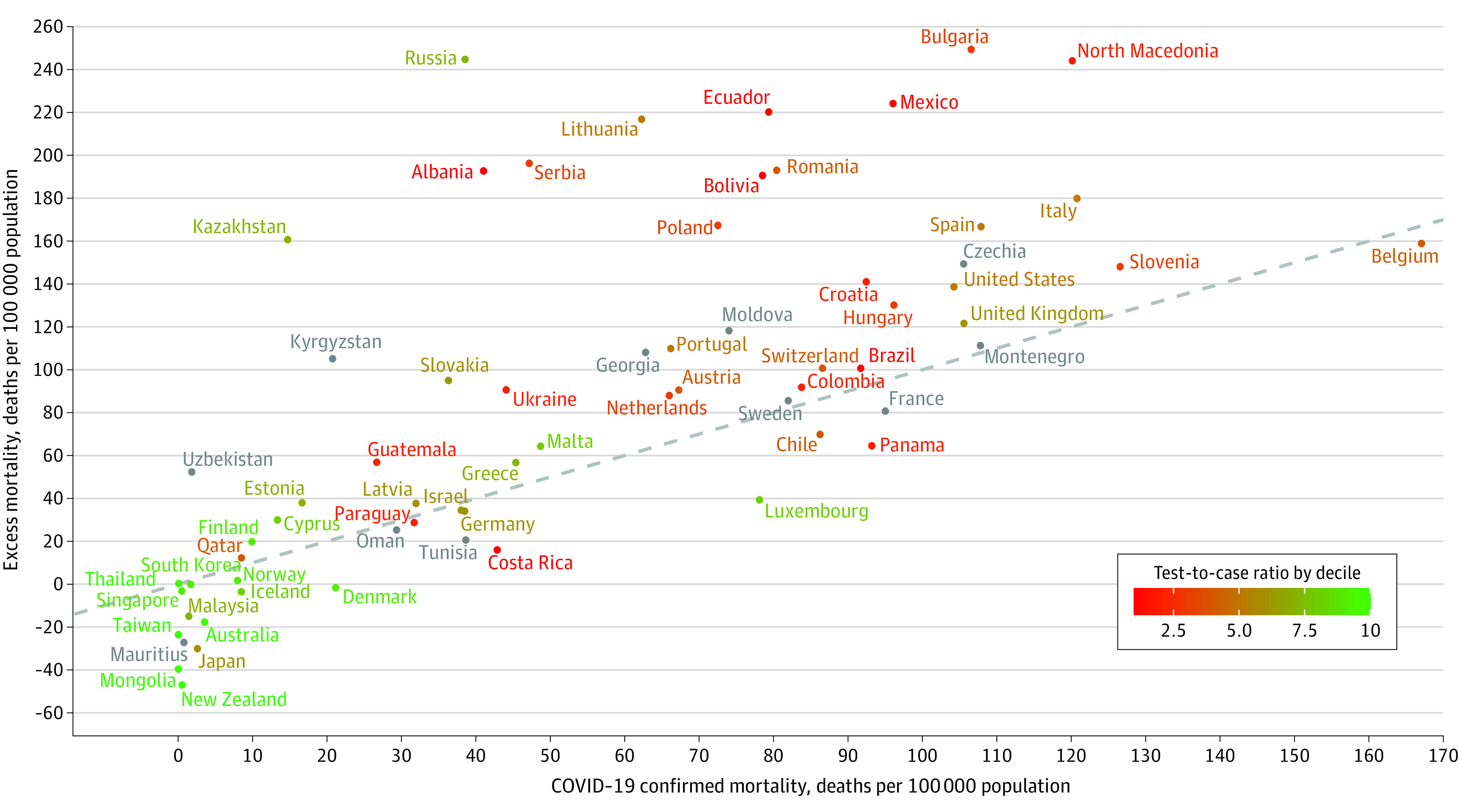
Scatterplot of COVID-19 Confirmed Mortality vs Excess Mortality in 67 Countries, February 26 to December 31, 2020 The dashed diagonal line represents the equality between the number of excess deaths and of COVID-19 reported deaths. The 0 marker on the y-axis indicates no excess mortality. Countries are colored according to their decile of the test-to-case ratio. Countries appearing in gray had unavailable or incomplete data on testing.

## Discussion

This comparison of CCM and EM revealed the different national health systems’ capacity to test and diagnose COVID-19 and their responsiveness to the health crisis. Underreporting of COVID-19 deaths because of strained health care systems’ capacity might explain our findings for countries where EM exceeded CCM.^2,4^ In contrast, the effects of nonpharmaceutical interventions on populations’ main causes of deaths, such as the decrease in work and road accidents, could be responsible for the reduction in overall mortality in countries where CCM exceeded EM.^5^ Notably, most of the countries that presented reduced overall mortality during 2020 had extremely high testing capacity and were praised for their effective response measures against the pandemic.^6^

Limitations of our analysis include the lack of stratification by age and sex, the underrepresentation of some areas of the world, and not considering nonpharmaceutical interventions. Despite these drawbacks, our findings corroborate the evidence that in many countries the accuracy in quantifying the death toll of COVID-19 is still a missed target. The global action against the pandemic is being conditioned by diverse responses to the crisis, but reliable evidence should be the pillar on which effective prevention measures are built.

## Notes

Supplement. **eAppendix.** Supplemental Methods
