# Supplementary material for: Exploring the Gap Between Excess Mortality and COVID-19 Deaths in 67 Countries
Source: JAMA Netw Open. 2021 Jul 16;4(7):e2117359. doi: 10.1001/jamanetworkopen.2021.17359 (PMC8285734; doi:10.1001/jamanetworkopen.2021.17359)
Supplement: Supplement. — eAppendix. Supplemental Methods [file jamanetwopen-e2117359-s001.pdf]

Supplemental Online Content

Sanmarchi F, Golinelli D, Lenzi J, et al. Exploring the gap between excess mortality and COVID-19 deaths in 67 countries. *JAMA Netw Open*. 2021;4(7):e2117359. doi:10.1001/jamanetworkopen.2021.17359

eAppendix. Supplemental Methods

This supplemental material has been provided by the authors to give readers additional information about their work.

© 2021 Sanmarchi F et al. *JAMA Network Open.*

eAppendix. Supplemental Methods

Data on overall deaths for each country from 2015 to 2020 were downloaded on April 6, 2021 from multiple official sources collected in the World Mortality Dataset[1] (Eurostat, Human Mortality Database, and National Statistics Offices). We included all countries that had at least 4 years of data released at weekly or monthly intervals, or at least 3 years at weekly intervals, in order to obtain our estimates of expected deaths using at least 48 time points (n=75 countries).

We decided to analyze data starting from 26 February 2020, given that deaths with COVID-19 up to that date were extremely rare (≤ 25 deaths) in the countries included in our analysis. Had we started our analysis before, the excess mortality estimates would have included an interval in which the COVID-19 pandemic had substantially no effect on overall mortality.

We retrieved data on COVID-19 deaths, overall confirmed cases, testing and population from Our World in Data (link: https://ourworldindata.org/coronavirus)[2] accessed on April 2, 2021. We excluded four countries that had no reported COVID-19-related deaths in 2020 (n = 71). We also excluded Armenia and Azerbaijan due to their involvement in the 2020 Nagorno-Karabakh conflict, because quantifying war casualties in order to adjust their excess mortality (EM) estimates proved to be difficult (n = 69). We also decided to exclude two microstates (San Marino and Liechtenstein) due to their small population sizes. As a result, the analysis was conducted on 67 countries, among which 42 had weekly overall mortality data and 23 had monthly overall mortality data.

Expected overall mortality of 2020 was estimated using a generalized negative binomial regression model. This model is a version of the negative binomial model in which the

[1] Karlinsky, A., Kobak, D., 2021. The World Mortality Dataset: Tracking excess mortality across countries during the COVID-19 pandemic. medRxiv 2021.01.27.21250604.
[2] Roser M, Ritchie H, Ortiz-Ospina E, et al. Coronavirus Pandemic (COVID-19). Published online at OurWorldInData.org 2020 https://ourworldindata.org/coronavirus

© 2021 Sanmarchi F et al. *JAMA Network Open.*

overdispersion parameter is modelled as a function of other variables, thus improving the fit to the data. The count of deaths was the outcome variable; the week (month) initial date and the week (month) number were included as explanatory variables in order to account for seasonality. EM was computed by subtracting the cumulative expected deaths from the cumulative observed deaths for each country. COVID-19 confirmed mortality (CCM) was determined as the weekly or monthly cumulative COVID-19 mortality for the 67 countries, according to the reported frequency of overall COVID-19 deaths. Test-to-case ratio for each country was computed using COVID-19 cumulative confirmed cases and tests performed up to December 31, 2020 and used to define countries' testing capacity. We categorized each country based on its decile of test-to-case ratio distribution. Country-specific CCM and EM were divided by the resident population in 2020 (per 100 000) and illustrated using a scatter plot, in which countries are colored according to the deciles of their test-to-case ratio as of December 31, 2020 (available for 56 out of 67 countries). All analyses were conducted using R version 4.0.4.
